# Genome-wide identification and expression analysis of trihelix transcription factor family in cucumber (*Cucumis sativus* L.) and their roles in biotic stress responses

**DOI:** 10.1186/s12864-025-12341-y

**Published:** 2025-11-20

**Authors:** Tianbao Xie, Xiaomeng Xue, Liang Chen, Ying Wang, Xinxin Geng

**Affiliations:** 1 Hubei Key Laboratory of Edible Wild Plants Conservation and Utilization, Hubei Normal University, Huangshi, 435002 China; 2 College of Life Sciences, Hubei Normal University, Huangshi, 435002 China; 3 Hubei Engineering Research Center of Characteristic Wild Vegetable Breeding and Comprehensive Utilization Technology, Huangshi, 435002 China

**Keywords:** *Cucumis sativus* L., Trihelix, Transcription factor, Gene structure, Stress responses

## Abstract

**Background:**

The Trihelix transcription factor family, characterized by a unique triple-helix structure, plays important roles in plant growth, development, and responses to biotic stresses. Cucumber (*Cucumis sativus* L.), a globally important horticultural crop, suffers significant growth and yield losses under biotic stresses (e.g., pathogens, insects, nematodes). Although Trihelix transcription factors have been well characterized in model plants like *Arabidopsis thaliana*, systematic analyses in cucumber are still limited. This study aims to comprehensively identify the Trihelix transcription factor gene family in cucumber using bioinformatics methods and analyze their expression patterns under biotic stresses, thereby providing insights into their potential roles in cucumber growth, development, and stress responses.

**Results:**

Using Arabidopsis Trihelix sequences as queries, we identified 29 CsaTri genes in *Cucumis sativus* L. cv. Chinese Long v4.0 genome (all containing the canonical Trihelix domain, named *CsaTri1*–*CsaTri29* by chromosomal position). Phylogenetic analysis classified these genes into 5 subfamilies (GT-1, GT-2, SH4, GT-γ, and SIP1), and they are unevenly distributed across 7 chromosomes. Gene structure and functional motif analyses revealed highly conserved motifs and domain architectures within the same subfamily. Cis-regulatory element analysis indicated that these genes may be involved in hormone responses, stress responses, and growth and development processes. Tissue expression analysis showed that *CsaTri12/CsaTri27/CsaTri28/CsaTri29* are highly expressed in stems, while *CsaTri8/CsaTri21* are highly expressed in roots. Transcriptome data and qRT-PCR results indicated that *CsaTri* genes actively respond to pathogen stress. Co-expression analysis linked *CsaTri* genes to immune pathways (PAMP recognition via LRR-RLK, post-transcriptional regulation via RNA metabolism/ubiquitin-proteasome system).

**Conclusions:**

Our study provides the first systematic characterization of the *Cucumis sativus* L. Trihelix transcription factor family, encompassing genomic, structural, evolutionary, and expression features. The identification of stress-responsive genes (e.g., *CsaTri18*, SIP1 subfamily) and their associated regulatory networks provides valuable insights into the potential biotic stress defense mechanisms of *Cucumis sativus* L. These findings lay a foundation for future functional validation of key *CsaTri* genes and offer potential candidate targets for breeding stress-resistant cucumber varieties.

## Background

Transcription factors are a class of proteins that can bind to specific DNA sequences in the promoter regions of genes, thereby regulating the initiation and rate of gene transcription. They play a crucial role in plant growth and development, physiological metabolism, and the process of responding to various biotic and abiotic stresses [1]. In plant immune responses, transcription factors are also key regulatory elements. When plants are invaded by pathogens, the transcription factors in their bodies can coordinate the immune response by activating or suppressing the expression of a series of immune-related genes, enabling plants to better resist pathogen invasion [2].

The Trihelix transcription factor family is named for its characteristic triple-helix structure (helix-loop-helix-loop-helix), and its core sequence is 5′-G-Pu-(T/A)-A-A-(T/A)−3′. The members can bind to the light-responsive GT element in DNA and participate in plant light signal transduction, growth and development, and responses to environmental stresses [3]. GT elements are widely present in the promoter regions of light-responsive genes (e.g., *RBCS*, *CAB*) and stress-responsive genes (e.g., *RD29A*, *COR47*), which explains why the Trihelix family can simultaneously regulate light signal transduction and stress response in plants [4]. Based on the domains and other sequence characteristics, the Trihelix transcription factor family can be classified into five families (GT-1, GT-2, GT-γ, SH4, and SIP1). Unlike other subfamilies, the GT-2 subfamily contains two DNA-binding domains, while the remaining subfamilies each possess one DNA-binding domain [5]. According to their target elements, Trihelix members can also be grouped into three classes (GT-1, GT-2, and GT-3): The GT-1 class factors specifically bind to the Box II (5′-GTGTGGTTAATATG-3′) and the 5′-GTTAC-3′ motif. The GT-2 class factors exhibit unique binding properties: their N-terminal binds to the GT-3 box (5′-GAGGTAAATCCGCGA-3′), while the C-terminal binds to the GT-2 box (5′-GCGGTAATTAA-3′). The GT-3 class factors also bind to the Box II (5′-GTGTGGTTAATATG-3′) and the 5′-GTTAC-3′, sharing binding elements with the GT-1 class factors. However, differences in specific gene regulation and biological functions remain unclear and require further investigation [6].

In Arabidopsis, 30 members of the Trihelix transcription factor family have been identified and divided into five subfamilies: GT-1, GT-2, SH4, GT-γ, and SIP1. Their functions involve a variety of plant physiological processes [7]. For example, GTL1 regulates leaf trichome development in Arabidopsis [8], *AST1* enhances drought and salt tolerance [9], and *HRA1* balances transcriptional activation of the hypoxic response during oxygen deprivation [10]. In tomato, 36 Trihelix family members have been identified [11]. Among them, *SlGT31* positively regulates fruit ripening. Inhibiting *SlGT31* expression delays ripening, reduces total carotenoid accumulation, decreases ethylene content, and suppresses the expression of ethylene- and ripening-related genes [12]. *ShCIGT* (GT-1 subfamily) is induced by abiotic stresses and abscisic acid; its overexpression enhances cold and drought tolerance, with transgenic plants showing reduced abscisic acid sensitivity during post-germination growth [13]. In rice, *OsGTγ−2* improves salt tolerance by regulating the expression of stress-related genes [14, 15]. Most of the research on the Trihelix transcription factor family has focused on salt/pathogen stress responses, perianth organ development, trichome/stomata formation, seed abscission layer, and late embryogenesis-related regulation. However, studies have also revealed their roles in abiotic stresses. In Arabidopsis, *ASR3* (Arabidopsis SH4-related 3) acts as a transcriptional repressor, negatively regulating pattern-triggered immunity (PTI). Its interacting partner, AITF1, forms heterodimers/homodimers with *ASR3* to co-regulate immune gene expression and resistance in a dependent manner [16]. Pathogens can exploit Trihelix factors for parasitism: the *Mi2g02* effector (secreted by the southern root-knot nematode) interacts with the host *GT-3a* in the nucleus, promoting root-knot nematode infection [17].

Cucumber (*Cucumis sativus* L.) is an annual trailing or climbing herbaceous plant belonging to the Cucurbitaceae family. As an important vegetable crop, it is widely cultivated worldwide and has significant economic and nutritional value [18]. However, due to its relatively delicate growth characteristics, it constantly faces a series of challenges throughout its growth cycle, including biotic stresses (e.g., pests and diseases) and abiotic stresses (e.g., drought, salinity, and extreme temperatures) [19]. Current research on the Trihelix transcription factor gene family in *Cucumis sativus* L. remains limited. Although some studies have explored certain aspects of *Cucumis sativus* L.‘s response to biotic and abiotic stresses, the specific roles and regulatory mechanisms of the Trihelix gene family in *Cucumis sativus* L. remain largely unclear.

In this study, we aimed to systematically analyze the Trihelix transcription factor gene family in *Cucumis sativus* L., focusing on molecular evolution, gene structure, cis-regulatory elements, conserved motifs, gene collinearity analysis, and protein three-dimensional structure prediction. This analysis will lay a solid foundation for exploring the functions of these genes in *Cucumis sativus* L. growth, development, and stress responses, ultimately providing a theoretical basis for cucumber molecular breeding.

## Methods

### Identification and physicochemical property analysis of Trihelix family members in *Cucumis sativus* L.

Genome annotation files, protein sequence files, and nucleic acid sequence files of *Cucumis sativus* L. cv. Chinese Long v4.0 were obtained from the Ensembl Plants database (http://plants.ensembl.org/index.html). Trihelix sequences of *Arabidopsis thaliana* and melon were used as query sequences for BLAST sequence alignment and HMMER searches [20]. Sequences with problematic domains or gene structures were excluded to identify the gene IDs of *Cucumis sativus* L. Trihelix gene family members and the amino acid sequences of their encoded proteins. The ProtParam module on the ExPASy website (https://web.expasy.org) were to analyze the physicochemical properties of the proteins encoded by *Cucumis sativus* L. Trihelix family members, including amino acid number, relative molecular weight, isoelectric point, instability coefficient, and hydrophobicity. Subcellular localization was predicted using the website WoLF PSORT website (https://wolfpsort.hgc.jp).

### Phylogenetic analysis and chromosome mapping

Amino acid sequences of the Trihelix family from *Arabidopsis thaliana* and melon were downloaded from the PlantTFDB website (https://planttfdb.gao-lab.org/index.php?sp=Ath). Amino acid sequences of *Cucumis sativus* L. and *Arabidopsis thaliana* Trihelix family genes were aligned using MEGA software, and a phylogenetic tree was constructed via the Maximum Likelihood method [21]. The tree was visualized and optimized using the iTOL website (https://itol.embl.de/itol.cgi) [22]. Chromosomal location information of *Cucumis sativus* L. Trihelix family genes was extracted from gene annotation files with TBtools software, followed by visualization to illustrate the relative positions of *Cucumis sativus* L. Trihelix genes on chromosomes [23].

### Three-dimensional protein structure modeling

The SWISS-MODEL website (https://swissmodel.expasy.org) was utilized to perform three-dimensional structure modeling of proteins encoded by the 29 *Cucumis sativus* L. Trihelix genes. Amino acid sequences of the proteins were submitted to the website, and upon obtaining the predicted 3D structures, the Global Model Quality Estimation (GMQE) scores provided by SWISS-MODEL were employed to assess model quality. Models with GMQE scores > 0.7 were selected, and Ramachandran plots were used to analyze amino acid residues distribution within the models. A model was considered to have a reasonable conformation if ≥ 90% of amino acid residues resided in the allowed regions of Ramachandran plot.

### Transmembrane domain prediction

Transmembrane domains of *CsaTri* Family Members were predicted using TMHMM Server v.2.0 (https://services.healthtech.dtu.dk/service.php?TMHMM-2.0). The tool generates probability distribution profiles across the entire protein sequence, with continuous regions of transmembrane probability > 0.6 and ≥ 18 contiguous amino acids annotated as potential transmembrane helices.

### Analysis of conserved motifs and domains

In TBtools software, gene IDs were used to query and search within gene annotation files, and combined with the phylogenetic tree to visualize the CDS and UTR sequences of *Cucumis sativus* L. Trihelix family genes. Conserved motifs of the *Cucumis sativus* L. Trihelix family were analyzed using the MEME online tool (https://meme-suite.org/meme/doc/meme.html). The maximum number of motifs to retrieve was set to 10, with other parameters maintained as default. These motifs were visualize using TBtools, and a combined diagram integrating the phylogenetic tree, gene structure, and conserved motifs was generated.

### Prediction of cis-regulatory elements and analysis of gene collinearity

Promoter sequence information was extracted from the *Cucumis sativus* L. genome data using TBtools and uploaded to the PlantCare website (https://bioinformatics.psb.ugent.be/webtools/plantcare/html/) for prediction of gene cis-regulatory elements. The website-returned files were processed to remove meaningless data and imported into TBtools software for visual mapping. All gene collinearity analyses were performed using TBtools software.

### Selective pressure analysis

The non-synonymous substitution rate (Ka) and synonymous substitution rate (Ks) of genes were calculated using DNaSP 6.0 software [24]. The selection pressure of duplicated gene pairs during evolution was evaluated by the Ka/Ks ratio (the submitted sequences were coding sequences): Ka/Ks > 1, < 1, and = 1 represent positive selection, negative selection, and neutral evolution, respectively. Visualization analysis was performed using R language [25]. The types of gene duplication were determined using the duplicate_gene_classifier tool in the downstream analysis program of MCScanX [26].

### Tissue-specific expression profile

The sequence information of target genes (members of the *CsaTri* family) and transcriptome data from different tissues (roots, leaves, flowers, fruits, stems, petioles, and cotyledons) were obtained from the *Cucumis sativus* L. Multi-omics Database (http://www.CucumissativusL.db.com/#/home), and Adobe Illustrator was used to combine the graphic information.

### Gene expression analysis under biotic stress

Based on transcriptome data from the website http://www.CucumissativusL.db.com/#/eFP, gene expression profiles of *Cucumis sativus* L. were obtained at different time points following treatments with ALS, CC, PM and GM. The CC treatment time points were 0, 6, 24, and 72 h; GM treatment included 0, 6, 12, and 48 h; PM treatment covered 0, 1, 3, and 5 days; and ALS treatment spanned 0, 1, 2, and 4 days. Differential expression analysis was performed using edgeR in R, calculating Log2FC (log2 fold change) values by comparing gene expression levels against the pathogen stress-free control. A heatmap was generated to visualize expression patterns of Trihelix family genes under distinct pathogen stresses and time points, where color intensity represented expression levels-red for up-regulation and blue for down-regulation.

### RNA extraction and quantitative real-time PCR

Cucumber leaves at the two-leaf-one-heart stage with vigorous growth were selected, and 6 leaf discs were uniformly punched from each leaf. These leaf discs were rehydrated in sterile water for 12 h to eliminate the interference caused by mechanical damage. Subsequently, the leaf discs were treated with 200 nM chitin solution and 200 nM flg22 solution, respectively. Total RNA was extracted from the leaf disc tissues using an RNA extraction kit (Vazyme Biotech Co., Ltd., Nanjing, China), followed by RNA quality detection using a NanoDrop 2000 micro-spectrophotometer (Thermo Fisher Scientific, USA). First-strand complementary DNA (cDNA) was synthesized with a reverse transcription kit (Vazyme Biotech Co., Ltd., Nanjing, China). For qRT-PCR analysis, the *CsaEF1* gene was used as the reference gene, and the relative expression levels of target genes were calculated by the 2^⁻ΔΔCT^ method. Three biological replicates were set for each qRT-PCR assay [27].

### Co-expression analysis of Trihelix gene family in *Cucumis sativus* L

In the co-expression module of the website http://www.CucumissativusL.db.com/#/coexpression, the Pearson Correlation Coefficient (PCC) screening threshold was set to ≥ 0.9 to identify significant co-expression relationships. The Sankey diagram was generated using https://www.chiplot.online/sankey_plot.html to visualize the co-expression network. In the Sankey diagram, the width of connecting lines represented the magnitude of co-expression correlation coefficients—wider lines indicated stronger co-expression relationships.

## Results

### Identification of the Trihelix transcription factor gene family in*Cucumis sativus* L.

To identify Trihelix family members in the *Cucumis sativus* L. genome and explore their characteristics and potential functions, we first referred to the known Trihelix protein sequences in *Arabidopsis thaliana* as queries, then performed the identification against the *C. sativus* L. cv. Chinese Long v4.0 (CLv4.0) reference genome. As a result, we successfully identified 29 Trihelix family members (Table 1). Each of these genes was verified to contain the Trihelix domain, and they were named*CsaTri1-CsaTri29* sequentially based on their chromosomal positions.

**Table 1:** Analysis of physical and chemical properties of trihelix gene family

Rename ID	CLv4.0 Accession ID	Amino Acid	MW (KDa)	pI	Genome Location	Subcellular Localization
CsaTri1	CsaV4_1G000070	910	100.62	8.37	Chr1	chlo^a^
CsaTri2	CsaV4_1G001665	642	72.87	5.92	Chr1	nucl^b^
CsaTri3	CsaV4_1G002723	310	34.87	6.07	Chr1	nucl
CsaTri4	CsaV4_1G002725	301	33.98	6.08	Chr1	nucl
CsaTri5	CsaV4_1G003894	618	71.11	6.06	Chr1	nucl
CsaTri6	CsaV4_2G001626	499	57.25	5.55	Chr2	nucl
CsaTri7	CsaV4_2G002547	398	45.52	6.09	Chr2	nucl
CsaTri8	CsaV4_3G001119	366	40.21	9.33	Chr3	chlo
CsaTri9	CsaV4_3G001721	960	108.35	6.6	Chr3	nucl
CsaTri10	CsaV4_3G003284	307	36.27	6.99	Chr3	nucl
CsaTri11	CsaV4_3G003593	547	61.55	5.9	Chr3	extr^c^
CsaTri12	CsaV4_3G003594	538	59.38	6.45	Chr3	nucl
CsaTri13	CsaV4_3G003620	517	59.15	6.61	Chr3	nucl
CsaTri14	CsaV4_3G004714	311	34.92	4.88	Chr3	nucl
CsaTri15	CsaV4_4G000705	274	32.71	7.71	Chr4	nucl
CsaTri16	CsaV4_4G001949	443	50.13	6.81	Chr4	nucl
CsaTri17	CsaV4_4G002174	293	35.31	8.39	Chr4	mito^d^
CsaTri18	CsaV4_5G000127	738	81.56	5.16	Chr5	nucl
CsaTri19	CsaV4_5G001045	410	46.57	8.63	Chr5	nucl
CsaTri20	CsaV4_5G003000	409	46.66	6.88	Chr5	nucl
CsaTri21	CsaV4_5G003134	345	38.56	9.92	Chr5	nucl
CsaTri22	CsaV4_6G000407	563	63.41	5.9	Chr6	nucl
CsaTri23	CsaV4_6G000408	653	73.32	5.9	Chr6	nucl
CsaTri24	CsaV4_6G000531	449	51.52	6.22	Chr6	nucl
CsaTri25	CsaV4_6G000791	355	38.74	5.48	Chr6	cyto^e^
CsaTri26	CsaV4_7G000497	263	30.63	9.44	Chr7	nucl
CsaTri27	CsaV4_7G001699	311	36.33	5.15	Chr7	mito
CsaTri28	CsaV4_7G002452	385	44.60	4.78	Chr7	nucl
CsaTri29	CsaV4_7G002561	373	40.96	9.67	Chr7	Nucl

^a^Chloroplast

^b^Nucleus

^c^Extracellular

^d^Mitochondria

^e^Cytoplasm

The proteins encoded by these Trihelix genes differ in length, ranging from 263 amino acids (CsaTri26) to 960 amino acids (CsaTri9). They also vary in molecular weight, with values ranging from 30.63 KDa to 108.35 KDa. Furthermore, their theoretical isoelectric points (pI) span a range, from 4.78 (CsaTri28) to 9.92 (CsaTri21). Through subcellular localization prediction, we found that 23 genes were predicted to be located in the nucleus, as evidenced by the relatively frequent annotation “nucl” (nucleus), which implies that many family members might function in the nucleus, potentially participating in nuclear-related biological processes like gene expression regulation. Additionally, certain proteins, such as *CsaTri1*, *CsaTri8*, *CsaTri17* and *CsaTri27*, were localized in chloroplasts or mitochondria, suggesting their possible involvement in specific metabolic functions and energy conversion within these organelles.

### Localization of chromosomes

**Fig. 1:**
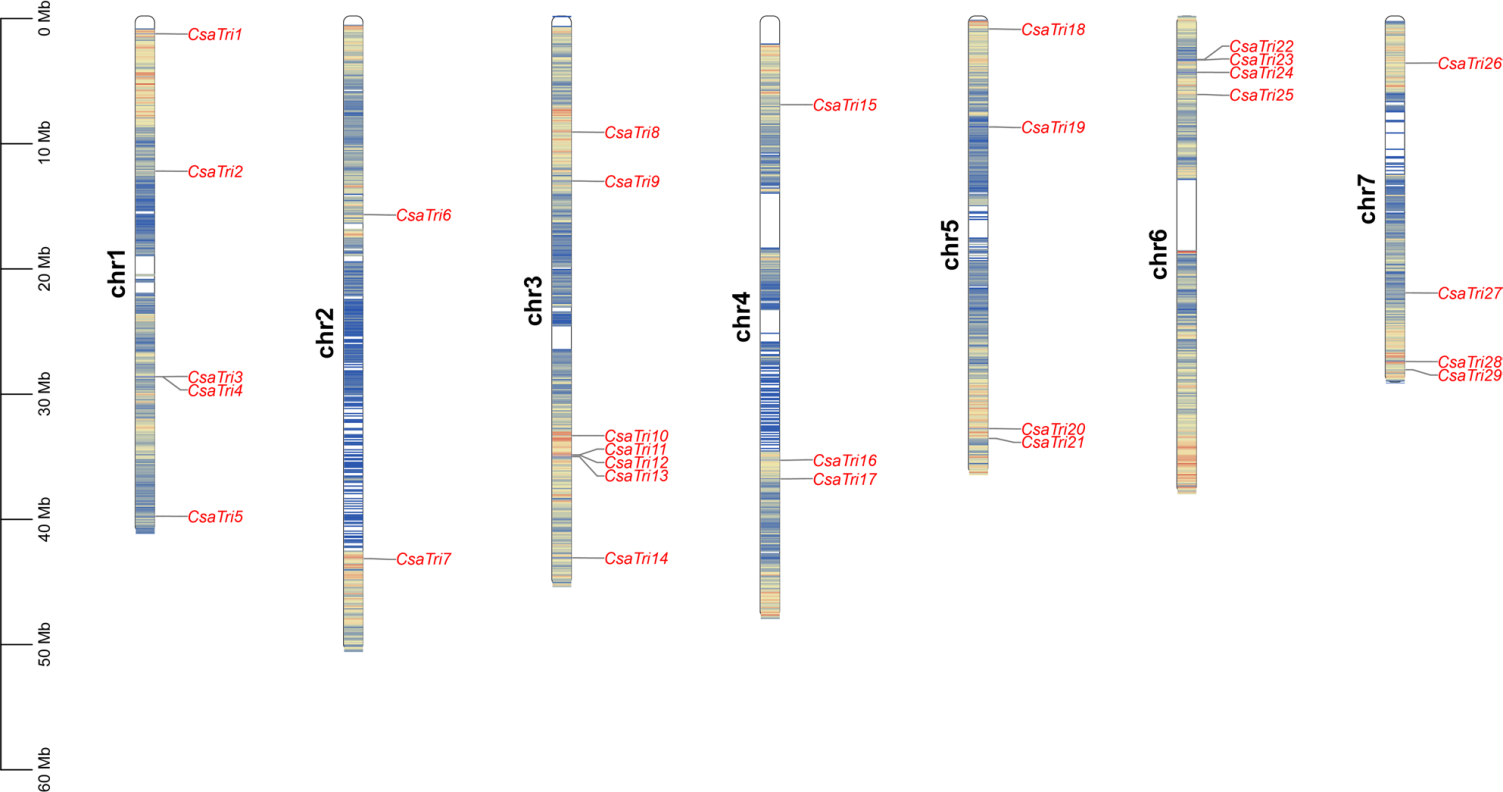
Distribution of the Trihelix family genes on *Cucumis sativus* L. chromosomes. The relative length of each chromosome indicates its size. The scale on the left denotes chromosome length in megabase pairs (Mb)

To visualize the chromosomal distribution of Trihelix transcription factor gene family members in *Cucumis sativus* L. and explore potential functional relationships, we generated Fig. 1, which depicts the location of 29 *Trihelix* genes across seven chromosomes. Colored stripes indicate gene density, revealing significant variation in gene numbers per chromosome; for instance, chromosome 3 harbors the highest number (seven genes), while chromosome 2 contains only two. Chromosome 5, 6 and 7 carried four Trihelix genes, and five Trihelix genes were located on chromosomes 1. Chromosome 4 carried three Trihelix genes. The results suggested a random distribution of the *Trihelix* genes among different chromosomes of *Cucumis sativus* L. As shown in Fig. 1, genes *CsaTri3-4*, *CsaTri11-13*, and *CsaTri22-23* exhibit highly concentrated positions. Our analysis suggests that these closely located genes may function synergistically, participating in shared or related biological processes. Presumably, during genome evolution, they were retained and evolved as a cohesive unit to execute specific functions, thereby forming a functional module.

### Structural characterization of the *Cucumis sativus* L. Trihelix gene family

**Fig. 2:**
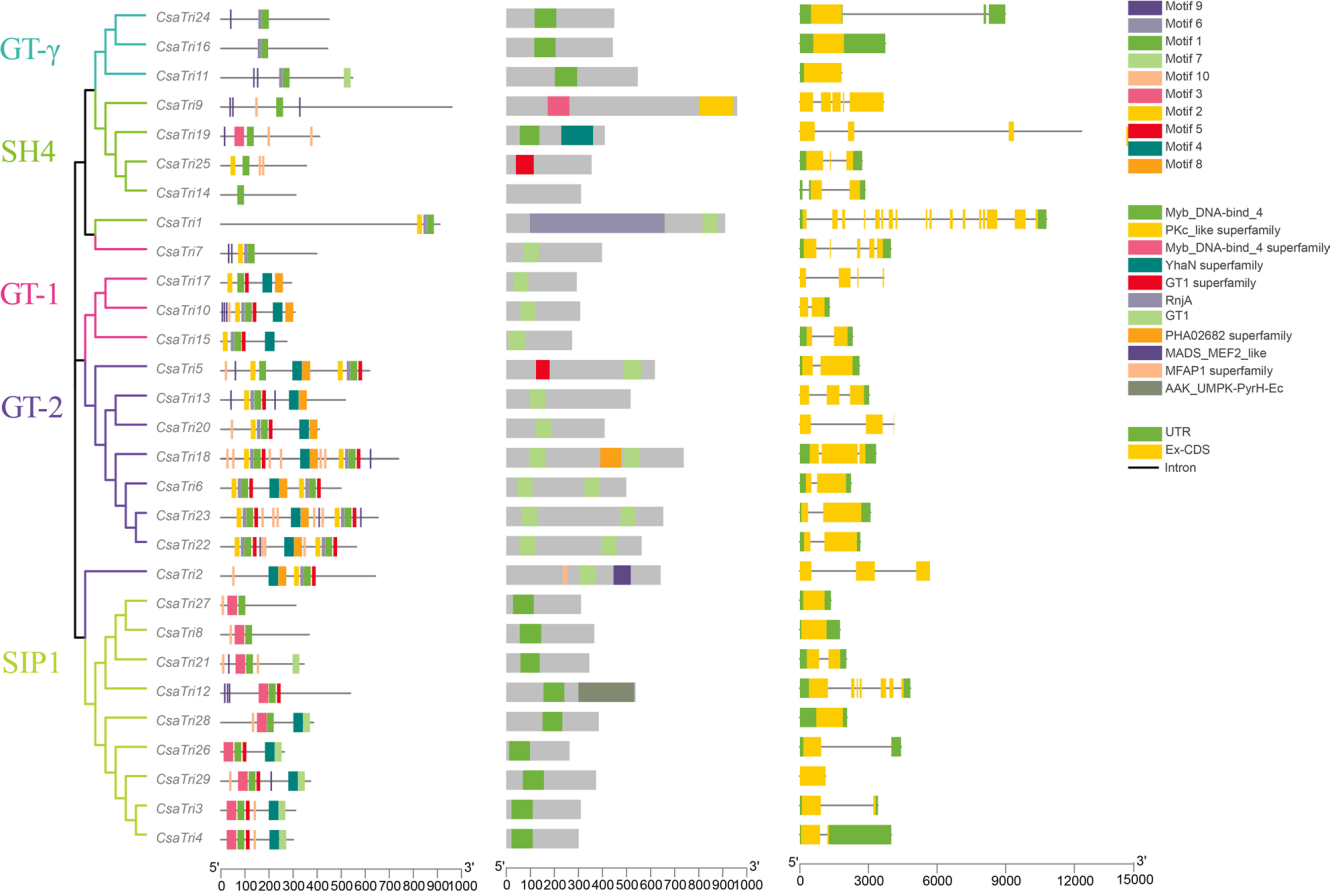
The phylogenetic tree on the far left classifies genes into different subfamilies (GT-γ, SH4, GT-1, GT-2, and SIP1) based on evolutionary relationships. Next, from left to right, are the following components: conserved motifs identified through MEME analysis, where distinct colors represent different motifs; conserved domains marked with different colors (e.g., Myb DNA bind); and the gene structures displayed on the far right, in which yellow boxes represent exons and black lines represent introns. The abscissa indicates the positions of base pairs (bp) and serves as a scale for measuring the length of each gene component

To identify and analyze the conserved motifs and structural characteristics of *Cucumis sativus* L. Trihelix genes, we employed the MEME Suite tool. As a result, ten conserved motifs were successfully identified, and their distributions are visualized in Fig. 2, demonstrating similar patterns within the same subfamily.

We found that all genes contained Motif 1, which overlapped with and resembled the helix-turn-helix structure of the MYB transcription factor. Thus, it was annotated as a Myb/SANT-like DNA-binding domain. The GT-1 subfamily all contain Motif 2. The GT-2 family specifically harbored Motifs 4, 5, 6, 8, and 10, with slight variations in their distribution positions. The GT-γ subfamily all contain Motif 6; the SH4 subfamily contain Motif 10 except for *CsaTri1* and *CsaTri14*; the SIP1 subfamily all contain Motif 3. Genes closely grouped in the phylogenetic tree shared similar motif characteristics; for example, *CsaTri8* and *CsaTri27*, *CsaTri3* and *CsaTri4*, belonging to the same topological structure, exhibited highly similar motif structures. These diverse motif arrangements and combinations contribute to the unique structures and functions of genes, indicating that specific motifs can serve as determinants for gene functions, facilitating gene classification and function prediction.

The analysis of the protein sequence structure diagrams revealed that the GT-1 and GT-2 subfamilies had the GT1 domain, while the GT-γ and SIP1 subfamilies contained the Myb_DNA-bind_4 domain. Members of the SH4 subfamily all contained different protein domain. The *CsaTri9* had the PKc_like superfamily, *CsaTri25* had the GT1 superfamily, while no protein domains were predicted in CsaTri14. Examination of gene structures showed that the number of exons in Trihelix family genes ranged from 1 to 17, with *CsaTri1* having the highest number (17 exons), consistent with the notion that genes with similar exon-intron structures often share evolutionary relationships.

### Phylogenetic analysis of the *Cucumis sativus* L. Trihelix gene family

**Fig. 3:**
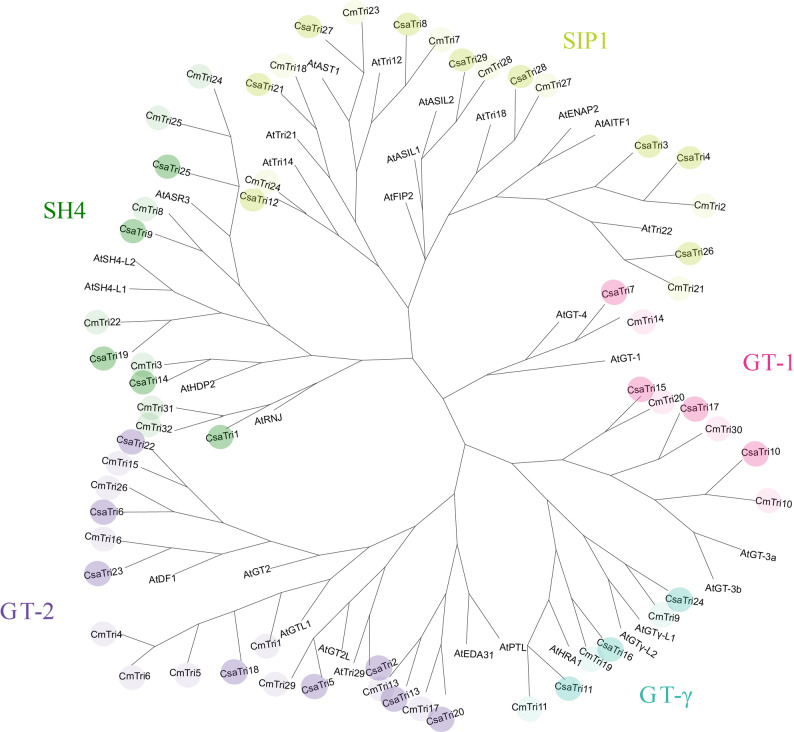
Phylogenetic relationships of Trihelix gene family members across species, including *Cucumis sativus* L., *Arabidopsis thaliana*, and *Cucumis melo* L. Clustering of Trihelix genes from these species into five subfamilies was achieved via phylogenetic tree construction

To elucidate the phylogenetic relationships among Trihelix proteins in *Arabidopsis thaliana* (At), *Cucumis melo* L. (Cm), and *Cucumis sativus* L. (Csa), a maximum likelihood phylogenetic tree was constructed using 91 Trihelix sequences. Specifically, 30, 32, and 29 sequences were sourced from *Arabidopsis thaliana*, melon, and *Cucumis sativus* L., respectively (Fig. 3). Among these species, the SIP1 subfamily, encompassing nine CsaTri members, represents the largest subfamily, whereas the GT-γ clade is the smallest, consisting of only three CsaTri members. The GT-2 and SH4 clusters contain eight and five CsaTri members, respectively. The phylogenetic tree revealed that SIP1 and SH4 exhibit a relatively close evolutionary relationship, as do GT-1 and GT-2. These findings suggest that genes within these subfamilies may share similar functions.

### Simulation of the three-dimensional structure of proteins

**Fig. 4:**
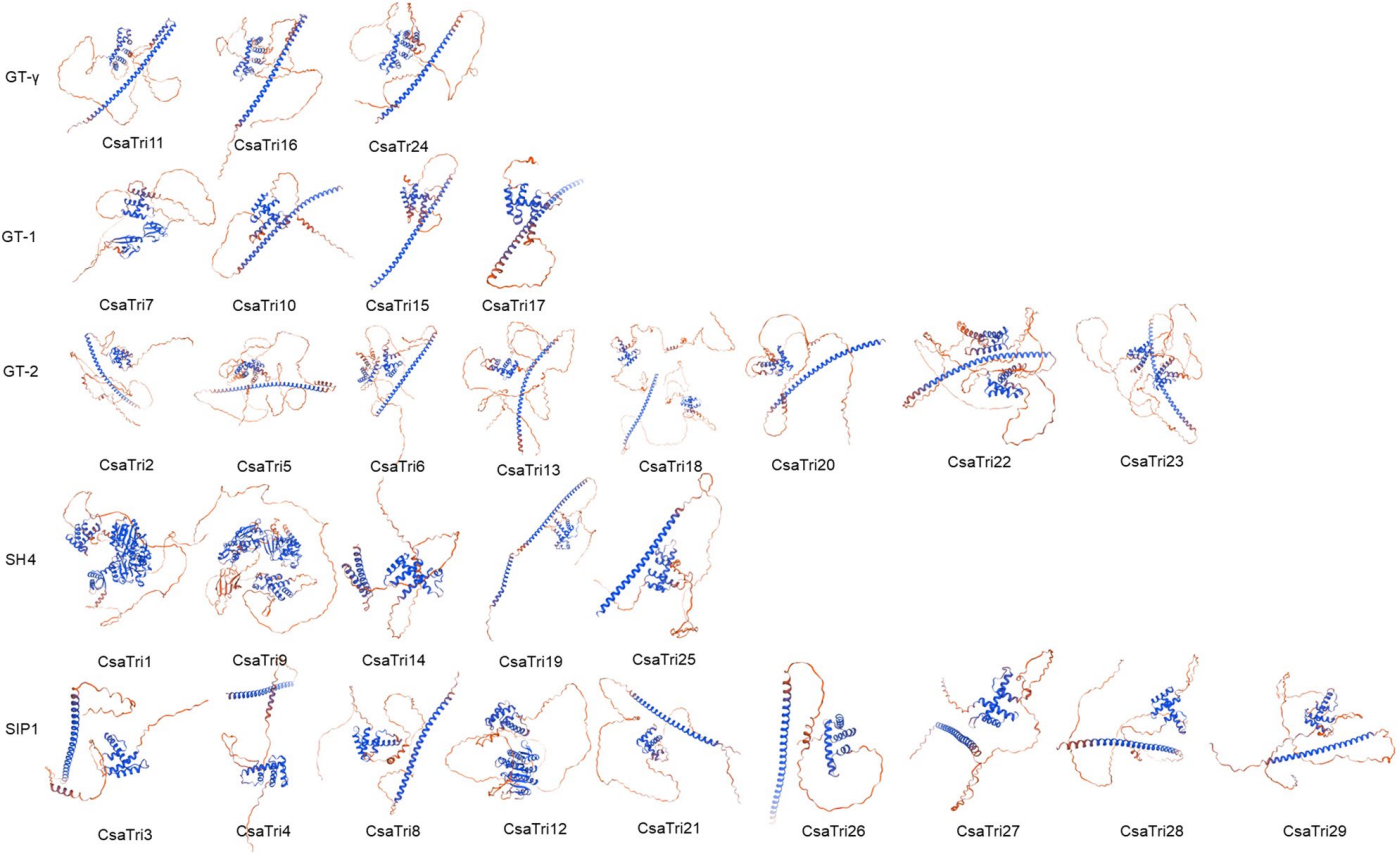
Three-dimensional structure modeling of *Cucumis sativus* L. Trihelix transcription factor family proteins. The 3D structures of 29 family members proteins were modeled using SWISS-MODEL

To elucidate the structural features and functional divergence of the Trihelix transcription factor gene family in *Cucumis sativus* L.s, we modeled the three-dimensional protein structures of all 29 family members using SWISS-MODEL. Model quality assessment revealed that all structures had GMQE scores > 0.7, and Ramachandran plots showed that > 90% of amino acid residues resided in allowed regions, validating the models’ reliability (Fig. 4).

Structural analysis indicated that all members contained the canonical helix-loop-helix-loop-helix motif, whereas the N- and C-terminal regions (residues XX–XX) exhibited substantial structural variation, potentially linked to functional specialization. Members of the GT-2 subfamily adopted more extended conformations with higher proportions of random coils compared to GT-1 subfamily members, suggesting enhanced flexibility that may facilitate interactions with diverse ligands. In the GT-γ subfamily, CsaTri11, CsaTri16, and CsaTri24 featured densely packed α-helical domains, forming well-ordered structural modules. SH4 subfamily members CsaTri1 and CsaTri9 displayed compact α-helical architectures with intricate interdomain linkages, likely optimized for mediating protein-protein interactions in signal transduction pathways. Conversely, SIP1 subfamily members such as CsaTri3 and CsaTri4 exhibited dispersed α-helical arrangements, distinct from other subfamilies in secondary structure organization.

### Transmembrane topology analysis

**Fig. 5:**
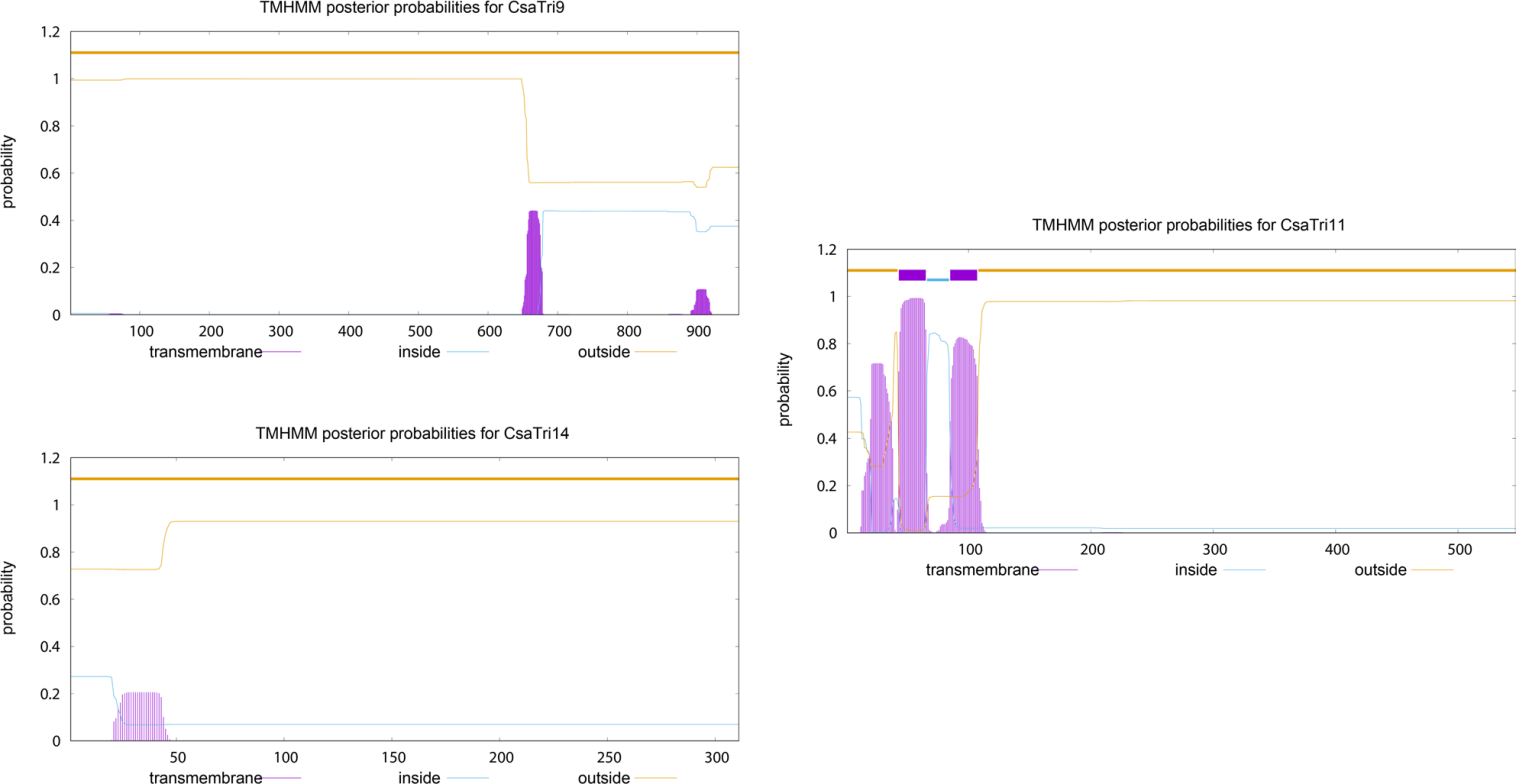
Posterior probability analysis by TMHMM revealed distinct transmembrane topologies and subcellular localization patterns among CsaTri9, CsaTri11, and CsaTri14, highlighting the divergence in their membrane protein structures and functional potentials

To investigate the membrane protein structures and functional potentials of CsaTri family members in cell membrane-related physiological processes, we performed TMHMM-based posterior probability analysis (Fig. 5). CsaTri9, CsaTri11, and CsaTri14 exhibited distinct transmembrane topology and subcellular localization patterns. CsaTri9 showed broad outside (orange) probability distribution with transmembrane (purple) and inside (blue) peaks in specific regions, indicating complex intra/extra-membrane organization despite its nuclear localization, potentially mediating nuclear-membrane signaling. CsaTri11 featured concentrated transmembrane (purple) peaks with minimal intra/extra-membrane probabilities, aligning with its extracellular localization and suggesting roles as a secretory or membrane-anchored protein in intercellular communication and transmembrane transport. CsaTri14 displayed initial transmembrane probability followed by dominant outside distribution, forming a unique membrane-associated pattern. These findings highlight divergent membrane protein architectures and functional potentials among CsaTri family members.

### Cis-regulatory elements analysis of the trihelix gene promoters

**Fig. 6:**
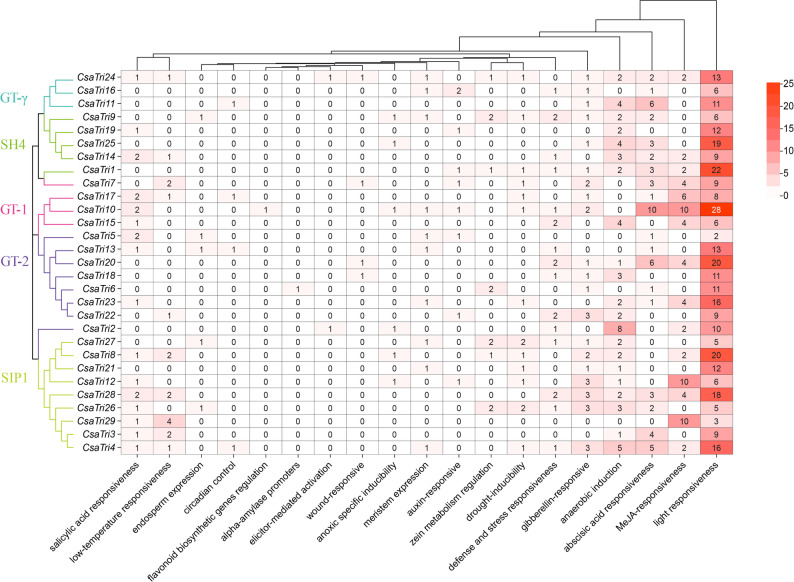
Promoter analysis of the *Cucumis sativus* L. Trihelix transcription factor gene family. Putative cis-regulatory elements exist in the 2000-bp upstream region of the *Cucumis sativus* L. Trihelix gene promoters

To explore the regulatory functions of *Cucumis sativus* L. Trihelix transcription factor gene family members, we extracted the 2000-bp sequence upstream of the transcription start site of each Trihelix gene as the promoter sequence. Functional prediction of cis-acting elements revealed a diverse array of cis-acting elements, reflecting the involvement of these genes in multiple biological processes (Fig. 6). Statistically, light responsiveness elements were the most abundant in Trihelix gene promoters — for instance, *CsaTri10* contained 28 such elements, *CsaTri1 22*, and *CsaTri8*/*CsaTri20* 20 each. This enrichment suggests that Trihelix genes may play conserved roles in mediating light-dependent growth and development in cucumber, aligning with their known functions in regulating photosynthesis-related genes and organ morphogenesis in other plants. Subfamily-specific patterns further highlight functional specialization: all GT-1 subfamily members harbored MeJA-responsive elements, while all SH4 subfamily members contained abscisic acid (ABA)-responsive elements. Given that MeJA and ABA are key hormones in plant defense signaling — MeJA primarily mediating responses to herbivory and necrotrophic pathogens, and ABA regulating abiotic stress tolerance and pathogen resistance — these findings imply that GT-1 and SH4 subfamilies may be preferentially involved in MeJA- and ABA-dependent stress pathways, respectively. In contrast, elements related to circadian control, flavonoid biosynthesis, and elicitor activation were rare, suggesting these processes are not primary targets of Trihelix-mediated regulation in cucumber.

In conclusion, the cis-acting elements of Trihelix transcription factor genes in *Cucumis sativus* L. are multifunctional, regulating complex biological processes such as growth, development, defense, and environmental adaptation in this species.

### Gene collinearity and selective pressure analysis

**Fig. 7:**
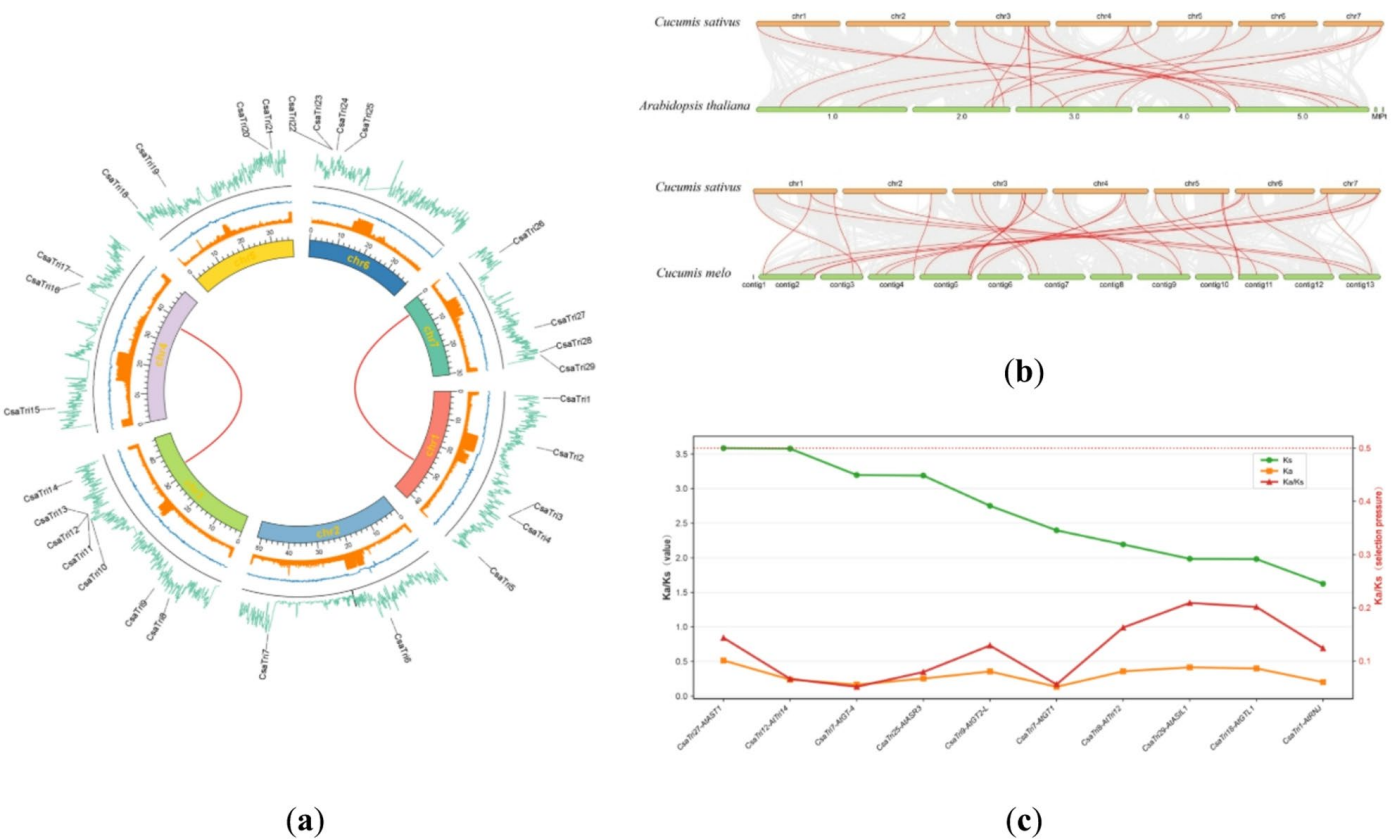
Collinearity analysis of *Cucumis sativus* L. Trihelix genes across multiple genomes. **a** Intragenomic collinearity analysis of the Trihelix gene pairs in *Cucumis sativus* L. The syntenic Trihelix gene pairs within the *Cucumis sativus* L. genome are connected by colored lines, with each color representing a distinct syntenic block. **b** Intragenomic collinearity analysis of the Trihelix gene between *Cucumis sativus* L. and Arabidopsis or melon. The syntenic Trihelix gene pairs between these genomes are depicted by grey lines. **c** Graph of Ka, Ks, and Ka/Ks trends for *Cucumis sativus* L.-*Arabidopsis thaliana* collinear gene pairs (sorted by Ks in descending order). Green line: Ks; orange line: Ka; red line: Ka/Ks

To gain deeper insights into the duplication mechanism of Trihelix gene family members in *Cucumis sativus* L., we constructed gene collinearity maps within the *Cucumis sativus* L. genome, as well as collinearity maps between *Cucumis sativus* L. and Arabidopsis, melon. Analysis revealed two pairs of collinear genes in the *Cucumis sativus* L. genome, namely *CsaTri17* and *CsaTri10; CsaTri26 and CsaTri4* (Fig. 7a). Additionally, the circos plot illustrates characteristics of the *Cucumis sativus* L. genome, with tracks from inside to outside representing GC content, unknown bases, and gene density.

The results of inter-species collinearity analysis show that 19 Trihelix genes in *Cucumis sativus* L. exhibit collinearity with 19 genes in *A. thaliana*; and 29 Trihelix genes show collinearity with 28 genes in melon (Fig. 7b). This indicates a relatively close genetic relationship between *Cucumis sativus* L. and melon.

To investigate the evolutionary patterns and functional correlations of duplicated gene pairs between *Cucumis sativus* L. and *Arabidopsis thaliana*, this study analyzed the variation trends of the Kₛ, Kₐ, and Kₐ/Kₛ ratio of these gene pairs (Fig. 7c). The results showed that: the Kₛ represented by the green line exhibited an overall decreasing trend, indicating that as the ranking of gene pairs progressed, the divergence time between the two genes in each pair gradually shortened; the Kₐ represented by the orange line was relatively stable overall, suggesting that the degree of functional differentiation among these gene pairs changed slightly; the Kₐ/Kₛ ratios represented by the red line were generally less than 1, demonstrating that these duplicated gene pairs were mainly subjected to negative selection pressure — specifically, during evolution, natural selection tends to retain mutations that can maintain the original functions of genes, thereby ensuring the stability and conservation of gene functions. From the perspective of gene function, in *Arabidopsis thaliana*, when plants are exposed to biotic stress, the *AtASR3*, *AtGTL1*, and *AtASIL1* genes can respond to pathogen stress; similarly, the functionally conserved *CsaTri25*, *CsaTri18*, and *CsaTri29* genes in *Cucumis sativus* may also be involved in similar disease resistance regulation processes.

### Expression profile of the Trihelix gene family in different tissues of *Cucumis sativus* L.

**Fig. 8:**
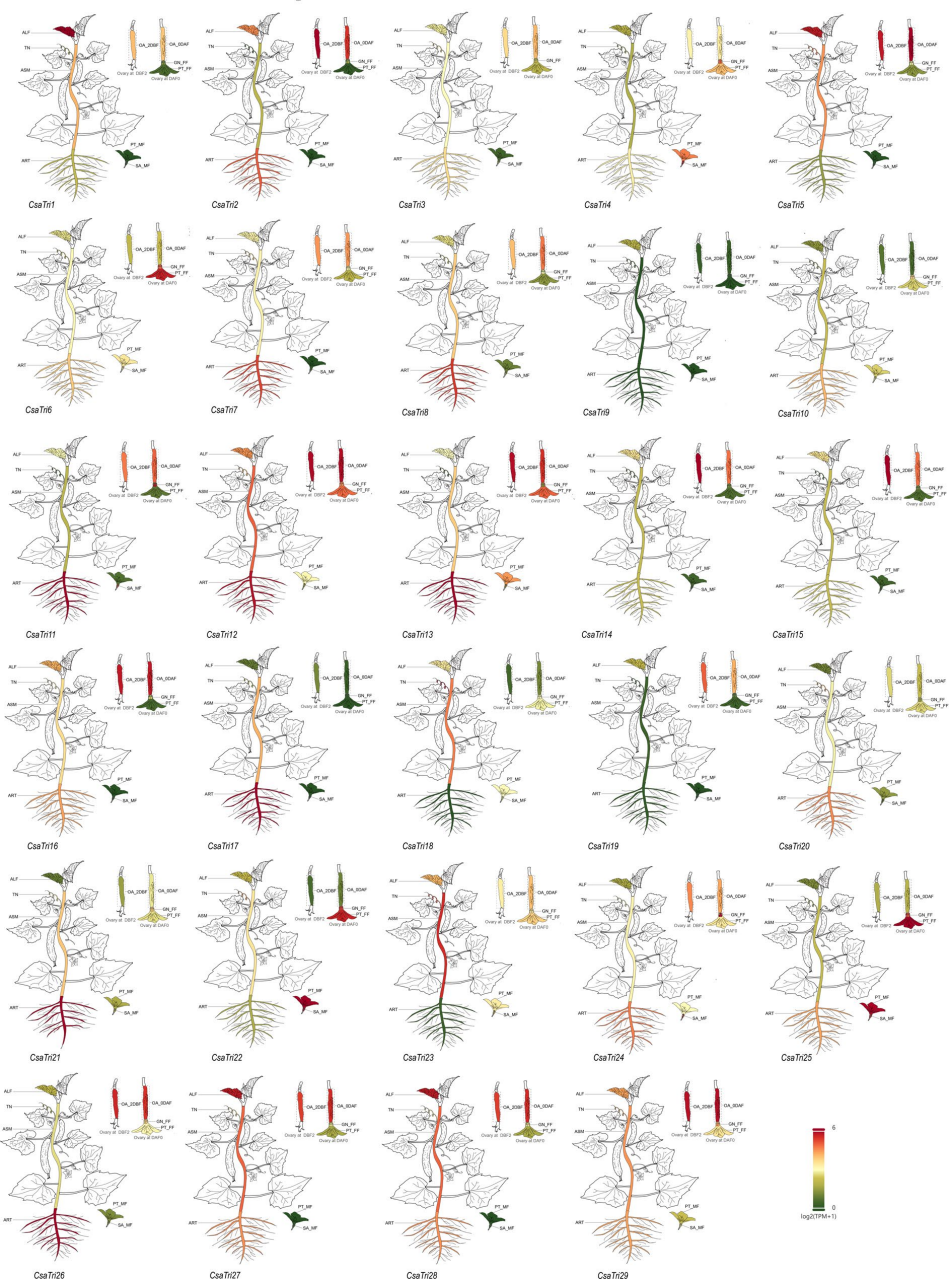
Expression profile of the Trihelix gene family in different tissues of *Cucumis sativus* L. The expression patterns of Trihelix genes across various *Cucumis sativus* L. tissues are visualized. Color gradients represent relative expression levels, with warmer colors (e.g., red) indicating higher expression and cooler colors (e.g., green) showing lower expression. Each plant schematic corresponds to a specific Trihelix gene, illustrating its transcriptional activity in roots, stems, leaves, flowers, and other tissues, providing insights into the tissue-specific regulation of the Trihelix gene family in *Cucumis sativus* L. ALF: Adult leaf; TN: Tendril; ASM: Adult stem; ART: Adult root; OA: Ovary; PT: Petal; SA: Stamen; MF: Male flower; FF: Female flower

To investigate the differential expression of Trihelix family genes in *Cucumis sativus* L. across various tissues, we conducted relevant analyses. The results revealed that *CsaTri11*, *CsaTri12*, *CsaTri13*, and *CsaTri17* are highly expressed in roots, a tissue frequently challenged by soil-borne pathogens. Additionally, *CsaTri1* and *CsaTri5* exhibit higher expression levels in leaves compared to other tissues, where foliar diseases such as powdery mildew commonly occur. Furthermore, *CsaTri12* shows elevated expression in flowers and fruits — organs critical for reproductive success and often targeted by pathogens like *Botrytis cinerea* — with its expression levels surpassing those in stems, petioles, and cotyledons. Conversely, *CsaTri3*, *CsaTri9*, *CsaTri10*, and *CsaTri20* generally display low expression levels across different tissues (Fig. 8). Overall, most Trihelix gene members in *Cucumis sativus* L. are expressed in various tissues, and their distinct expression patterns suggest potential roles in tissue-specific disease resistance.

### Response of the trihelix transcription factor family in *Cucumis sativus* L. to biotic stresses

**Fig. 9:**
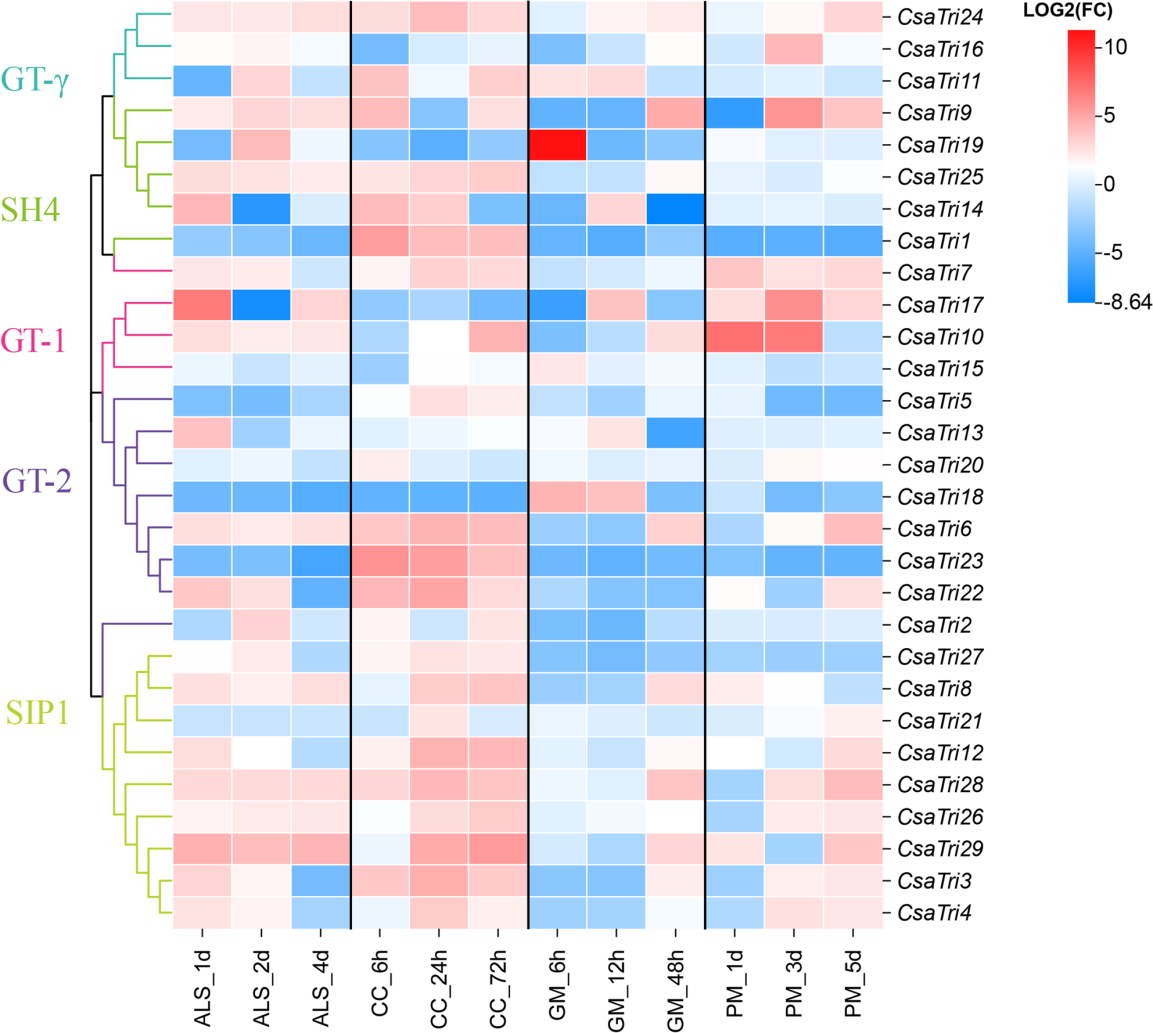
Heatmap of Trihelix gene expression patterns in *Cucumis sativus* L. under pathogen treatments. This heatmap depicts the expression profiles of *Cucumis sativus* L. Trihelix transcription factor genes at different time points following inoculation with four pathogens. Values represent log2 fold changes (Log2FC) in expression relative to untreated controls. Pathogens and treatment time points are as follows: CC: 0 h, 6 h, 24 h, 72 h; GM: 0 h, 6 h, 12 h, 48 h; PM: 0 d, 1 d, 3 d, 5 d; ALS: 0 d, 1 d, 2 d, 4 d. In the heatmap, red indicates upregulation, with darker red hues corresponding to higher Log2FC values. Conversely, blue indicates downregulation, with darker blue hues representing lower Log2FC values

To investigate the response mechanism of the Trihelix transcription factor family in *Cucumis sativus* L. to biotic stresses, this study analyzed the family’s gene expression heatmaps at different time points under treatments of ALS, CC, GM and PM using data from the SRA database. Results showed that under these pathogenic stresses, Trihelix genes exhibited diverse expression patterns (Fig. 9), reflecting their functional specificity in defense responses. Notably, *CsaTri24*, *CsaTri28*, and *CsaTri29* were significantly up-regulated under multiple stresses (ALS, CC, PM), while *CsaTri18* was significantly down-regulated under the same stresses. The SIP1 subfamily showed high coordination across all tested biotic stresses; this subfamily-wide induced expression may be an adaptive mechanism in cucumber—by synchronously activating SIP1 members, the plant may enhance the recognition of conserved PAMPs or amplify downstream defense signals, thereby improving broad-spectrum resistance to bacterial and fungal pathogens. In contrast, the GT-γ and SH4 subfamilies exhibited more complex expression patterns.

**Fig. 10:**
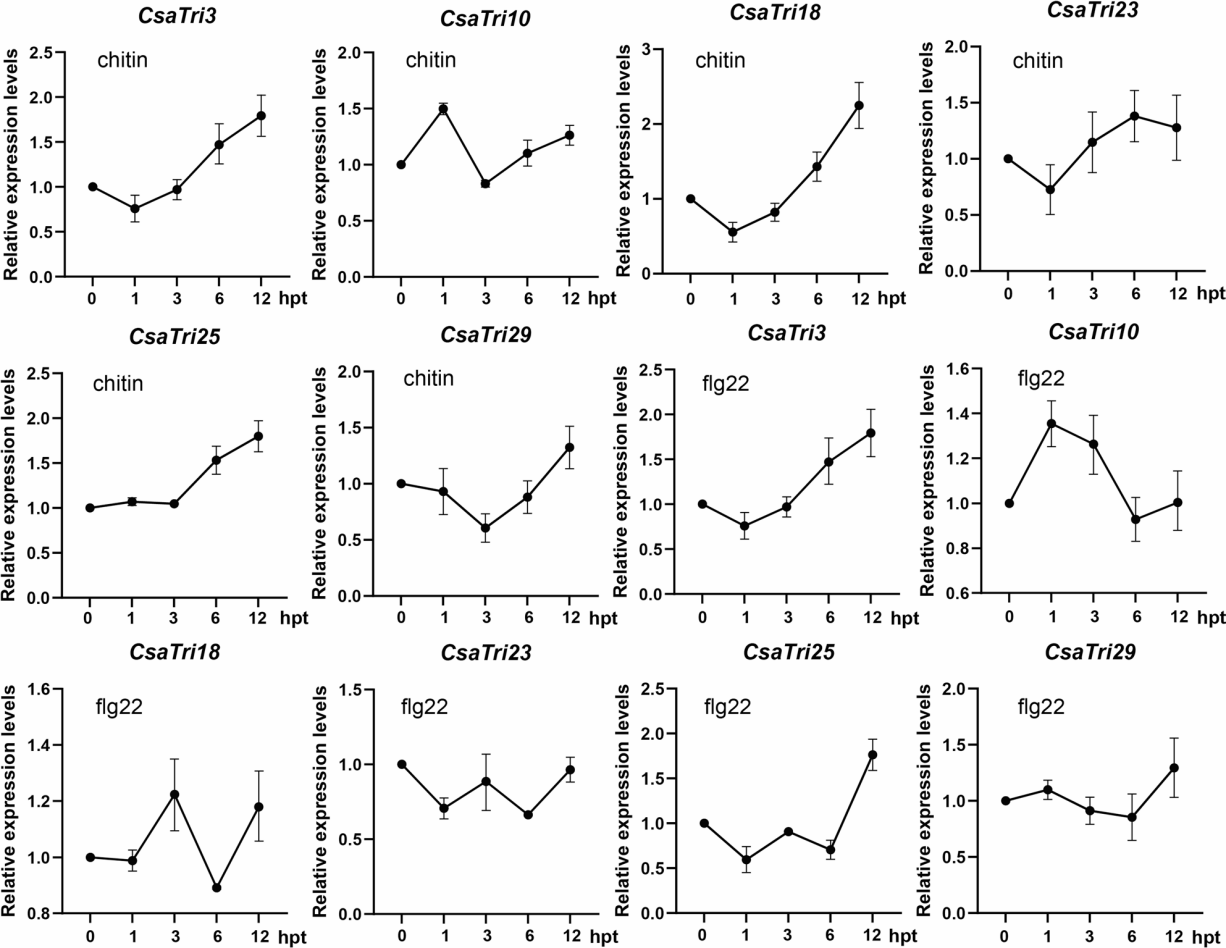
Expression patterns of *CsaTri* genes in leaves of *Cucumis sativus* L. at different time points under simulated pathogen infection treatments. The gene expression levels were determined at 0, 1, 3, 6, and 12 h post-treatment (hpt) with chitin and flg22. Error bars represent the standard error of three independent biological replicates

To further explore the transcriptional response of cucumber Trihelix genes to simulated bacterial/fungal infection, six representative genes (*CsaTri3*, *CsaTri10*, *CsaTri18*, *CsaTri23*, *CsaTri25*, *CsaTri29*) from each subfamily were selected based on the aforementioned basal expression patterns. Cucumber leaves were treated with chitin (for fungal infection simulation) and flg22 (for bacterial infection simulation), and the expression of these genes post-treatment was detected via qRT-PCR (Fig. 10). Under chitin treatment, most CsaTri genes showed defense-related differential expression: *CsaTri3*, *CsaTri18*, and *CsaTri28* exhibited “slow down-regulation in the early stage-sustained up-regulation in the late stage”, with *CsaTri3* and *CsaTri18* showing significant up-regulation in the late stage (consistent with the “signal perception - defense activation” feature of plants in response to fungal infection); *CsaTri10* was significantly up-regulated at 1 h post-treatment (potentially involved in early signal recognition), declined at 3 h, and gradually increased afterward; *CsaTri25* remained stable within 1–3 h, increased significantly from 6 h, and reached a high level at 12 h (potentially involved in late-stage defense or metabolic regulation). Under flg22 treatment, most CsaTri genes also showed differential expression, but their patterns were distinct from those under chitin treatment (reflecting pathogen-specific responses): *CsaTri3*, *CsaTri10*, and *CsaTri28* had similar trends to those under chitin treatment (potentially involved in common defense pathways, possibly related to the adaptive mechanism of SIP1 subfamily); *CsaTri18* and *CsaTri23* showed obvious fluctuations without stable up-regulation or down-regulation trends; *CsaTri25* and *CsaTri29* remained stable within 1–6 h and increased sharply at 12 h.

### Co-expression analysis of Trihelix gene family in *Cucumis sativus* L.

**Fig. 11:**
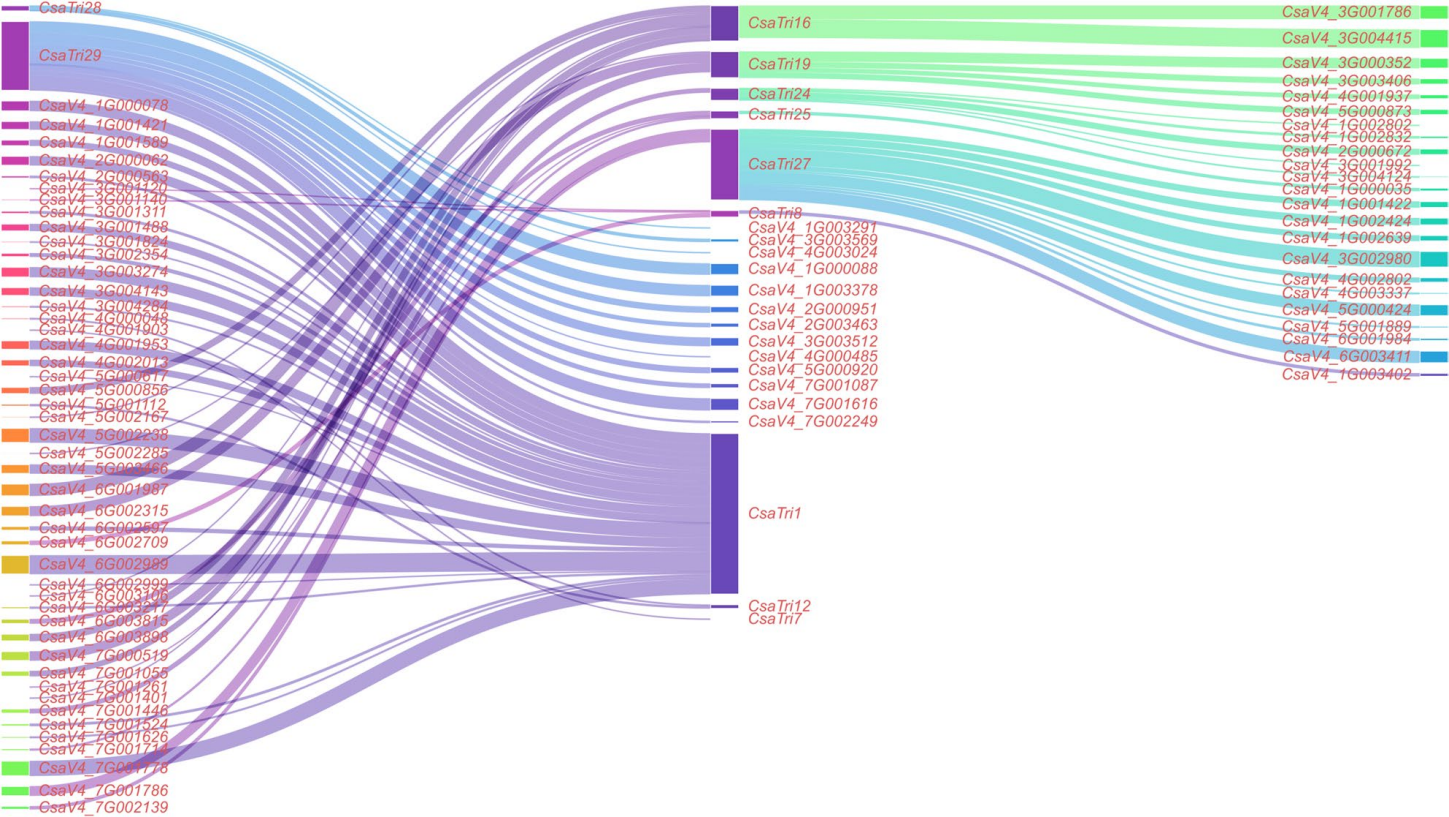
Sankey diagram of co-expression networks for *Cucumis sativus* L. Trihelix family genes. This diagram depicts co-expression relationships between *Cucumis sativus* L. Trihelix family genes and related functional genes. The width of the connecting lines represents the magnitude of the Pearson correlation coefficient (PCC), with wider lines indicating stronger co-expression relationship

To explore the synergistic mechanisms of *Cucumis sativus* L. Trihelix family genes in growth, development, and immune regulation, we performed co-expression analysis (Fig. 11). Results showed that *CsaTri19* co-expressed significantly with *CsaV4_3G003406* (encoding a kinesin-like protein) and *CsaV4_5G000873* (encoding LRR-RLK). The latter acts as a pattern recognitiobn receptor on plant cell membranes, crucial for recognizing PAMPs and activating downstream immune pathways. *CsaTri19* also co-expressed with family member *CsaTri25*, implying Trihelix transcription factors may synergize via regulatory networks in plant immunity. *CsaTri1* (encoding ribonuclease J) co-expressed with genes like *CsaV4_2G000563* (DNA-dependent RNA polymerase 3) and *CsaV4_3G001824* (pentatricopeptide repeat protein). These associations suggest ribonuclease J modulates plant immunity at the post-transcriptional level by regulating immune-related transcript processing/degradation, critical for maintaining RNA metabolic balance during pathogen invasion. Additionally, *CsaTri16* co-expressed with ubiquitin-proteasome system genes (e.g., *CsaV4_3G004415* encoding ubiquitin carboxyl-terminal hydrolase 14 and *CsaV4_1G002802* encoding ubiquitin-conjugating enzyme E2). The ubiquitin-proteasome system regulates plant immune signaling via targeted protein degradation, indicating these co-expressed genes may synergize to maintain protein homeostasis during immune responses.

## Discussion

The Trihelix transcription factor family, crucial for plant stress adaptation, has been systematically characterized in *Cucumis sativus* L. (based on the CLv4.0 genome), with 29 members unevenly distributed across seven chromosomes. A notable feature specific to cucumber is the highest gene density on Chromosome 3 (7 genes), including tandem clusters (e.g., *CsaTri11-CsaTri13*) inferred to originate from segmental duplication. This clustering differs from the dispersed distribution of Trihelix genes on Arabidopsis Chromosome 5 [28], while all three species rely on duplication for family expansion, cucumber’s Chromosome 3 enrichment may reflect a lineage-specific adaptation to its typical cultivation environments (e.g., high humidity-induced pathogen pressure), a pattern not reported in other Cucurbitaceae crops to date [29]. Structural analysis revealed that all cucumber Trihelix members harbor a conserved Myb/SANT-like DNA binding domain (Motif 1), consistent with the domain conservation observed in Arabidopsis and rice [30]. However, subfamily-specific motifs (e.g., Motif 4, 5, 10 in the GT-2 subfamily) are more diverse in *Cucumis sativus* L. than in Arabidopsis: cucumber GT-2 members contain 3 unique subfamily motifs, whereas Arabidopsis GT-2 only has 1 [31]. Three-dimensional modeling further uncovered cucumber-specific conformational traits: the GT-2 subfamily’s high proportion of random coil structures (≈ 40% of total secondary structure) is 15% higher than that in rice GT-2 proteins [32], which may enable more flexible binding to diverse cis-elements (e.g., overlapping GT and G-box motifs) in stress-responsive gene promoters. In contrast, the GT-γ subfamily’s α-helix-dominated structure (≈ 65%) aligns with rice homologs, suggesting functional conservation in maintaining stress response stability [33].

Promoter cis-acting element analysis highlighted cucumber-specific regulatory biases. While light-responsive motifs (e.g., G-box, ACE) are common in Trihelix promoters of both *Cucumis sativus* L. and rice [34], cucumber’s GT-1 subfamily uniquely enriches JA-responsive CGTCA-motifs (present in 80% of GT-1 members) — a proportion twice as high as in Arabidopsis GT-1 [35]. This suggests *Cucumis sativus* L. GT-1 may play a more prominent role in JA-mediated biotic stress responses (e.g., downy mildew resistance). Additionally, the SH4 subfamily in *Cucumis sativus* L. contains ABA-responsive ABRE elements (in 75% of members), similar to that in Arabidopsis (i.e., 75% of Arabidopsis SH4 homologs also contain ABRE elements) [36], implying conserved involvement in ABA-dependent abiotic stress (e.g., drought) regulation.

Transcriptome data under ALS, CC, GM, and PM infections further supported *Cucumis sativus* L.-specific functional divergence. *CsaTri9* and *CsaTri24* were consistently upregulated across all four pathogens (log2FC > 1.5), a broad-spectrum response not observed in Arabidopsis Trihelix genes (which typically respond to 1–2 pathogens). This suggests these two genes may act as core regulators of *Cucumis sativus* L.’s general immune response. Conversely, *CsaTri10* and *CsaTri29* were downregulated only during early CC infection (log2FC < −1.0), resembling the negative regulatory role of Arabidopsis ASR3 but with pathogen-specificity — unlike *ASR3*, which responds to multiple pathogens [37]. The SIP1 subfamily’s universal upregulation under all stresses aligns with Betula platyphylla homologs [38], while GT-γ and SH4 showed pathogen-specific expression (e.g., GT-γ upregulated only under PM), indicating subfamily-level functional differentiation unique to *Cucumis sativus* L. qRT-PCR results showed that genes such as *CsaTri3* and *CsaTri29* actively responded to pathogen stress, and members of the *Cucumis sativus* L. Trihelix family exhibited different expression profiles in response to bacterial and fungal biotic stresses.

Co-expression analysis identified potential *Cucumis sativus* L.-specific regulatory networks. *CsaTri19*’s association with kinesin-like proteins and LRR-RLKs (e.g., *CsaV4_5G000873*) has not been reported in Arabidopsis or rice, suggesting a novel mechanism in *Cucumis sativus* L. for regulating receptor trafficking during pathogen recognition [39]. *CsaTri1* (encoding ribonuclease J) co-expressed with RNA metabolism genes (e.g., DNA-dependent RNA polymerase 3) — a link analogous to that of cotton *GhGT-3b_A04*, but *Cucumis sativus* L.-specific in its role in post-transcriptional control of immune transcripts under biotic stress [40].

The direct interactions between CsaTri genes and their downstream targets, as well as the specific molecular mechanisms (e.g., via RNA binding or protein-protein interaction) underlying their potential role in post-transcriptional regulation of immune transcripts, remain unclear. Additionally, co-expression network analysis only identified potential gene associations without validating protein-level interactions (e.g., yeast two-hybrid, bimolecular fluorescence complementation), and the network’s reliability requires further empirical confirmation. Furthermore, the lack of association analysis between CsaTri sequence variations and stress resistance phenotypes across *Cucumis sativus* L. cultivars limits the accurate assessment of this gene family’s potential breeding value.

## Conclusions

This study systematically characterized the *Cucumis sativus* L. Trihelix transcription factor family via genomic, structural, evolutionary, and expression analyses, identifying 29 CsaTri genes with diverse features; phylogenetic/collinearity analyses revealed conserved and lineage-specific evolutionary patterns, while cis-acting element and expression analyses indicated their potential roles in light response, hormone signaling, and biotic stress defense—with the coordinated up-regulation of the SIP1 subfamily and co-expression networks with immune-related genes providing new insights into cucumber’s defense mechanisms. These findings have theoretical and practical reference value, as the standardized nomenclature and comprehensive characterization lay a foundation for future functional studies and the identification of stress-responsive genes (e.g., *CsaTri18*, SIP1 subfamily) provides potential candidates for cucumber disease resistance breeding, though limitations exist in that most conclusions rely on in silico predictions, necessitating experimental validation (e.g., gene knockout/overexpression, promoter-GUS assays) to confirm gene functions, with future research focusing on verifying key CsaTri genes’ roles in biotic stress responses and exploring their molecular regulatory mechanisms.

## Data Availability

The datasets used and analysed during the current study are publicly available in the Cucumber-DB repository, [http://www.cucumberdb.com/#/eFP].

## Abbreviations

ABA: Abscisic Acid
ABRE: ABA-Responsive Element
ALS: Angular Leaf Spot
BLAST: Basic Local Alignment Search Tool
CC: Cladosporium Cucumerinum
CDS: Coding Sequence
FC: Fold Changes
GM: Gray Mold
HMMER: Hidden Markov ModelER
JA: Jasmonic Acid
Ka: Non-synonymous Substitution Rate
Ks: Synonymous Substitution Rate
Ka/Ks: Ratio of Non-synonymous to Synonymous Substitution Rates
LRR-RLK: Leucine-rich Repeat Receptor-Like Kinase
MAP: Mitogen-Activated Protein
MEME: Multiple Expectation Maximization for Motif Elicitation
MPK: Mitogen-Activated Protein Kinase
MYB: MYB Transcription Factor Family
PAMP: Pathogen-Associated Molecular Pattern
PCC: Pearson Correlation Coefficient
pI: Isoelectric Point
PM: Powdery mildew
PTI: PAMP-Triggered Immunity
qRT-PCR: Quantitative Real-Time Polymerase Chain Reaction
RLK: Receptor-Like Kinase
SA: Salicylic Acid
SWISS: SWISS-PROT Protein Database
